# Anæsthesia vs. Asphyxia

**Published:** 1886-11

**Authors:** S. J. Hayes

**Affiliations:** Pittsburg, Pa.


AMERICAN JOURNAL

—OF—

DENTAL SCIENCE.

VOL. XX. THIRD SERIES—NOVEMBER, 1886. NO. 7.

ARTICLE I.

### ANÆSTHESIA VERSUS ASPHYXIA.

BY DR. S. J. HAYES, PITTSBURG, PA.

It is to be very much regretted that the vast difference between anæsthesia and asphyxia, has been almost entirely overlooked by writers on anæsthetics and artificial anæsthesia. And yet, not until, we are prepared to concede that anæsthesia is produced by the continuation of the *forces* and *functions* of life under modifications induced by medico -chemical agencies, and that asphyxia is the result of a suspenion or abrogation of these forces and functions,—also, that oxygen is the only *life giving principle*, then, and not, until then, can we fully recognize the *essential*, *fundamental and "bedrock* principle" that there can be no anæsthetic without a proper admixture of atmospheric air with a suitable narcotic, (except it be locally applied,) which must be necessarily and unchangeably true, as the atmosphere is the only element in the physical world that will sustain respiration and life and therefore is *absolutely essential* to an anæsthetic.

Though the gases composing the pure atmosphere, may be breathed in other relative proportions or other gases such as carbonic acid (C. O.2), carbonic oxide, (C. O.), or nitrous oxide, (N. O.), may be taken, for a very limited time, yet they do not support respiration and life and consequently, are not anæsthetics and are never taken without physical injury or positive danger to life. In fact, they antagonize life from the very first inspiration. True, they produce unconsciousness and insensibility, but by asphyxia which is the legitimate result of depriving the blood of the life giving principle,—oxygen—and thus the death of the patient is inevitable when the continuous use of the nitrous oxide or other gas, has so weakened the affinity of the blood for oxygen that it fails to appropriate the oxygen from the air. The failure of these gases to sustain life is not attributable to a lack of oxygen in them, as they contain a greater quantity of oxygen than the atmosphere; but, that the affinity of the oxygen for the other gas, is greater or stronger than the affinity of the blood for the oxygen and consequently, the blood cannot appropriate the oxygen, and therefore, the gas fails to support life.

And yet, a very common error, entertained by many, both in the medical and dental professions, is that as nitrous oxide (N. O.) consists simply of the elements of common air, (N. O.4) only in different proportions, that it certainly cannot do harm and that since it has a larger proportion of the life giving principle—oxygen—when inhaled it should only make one live faster, than when he breathes the air. But this shows gross ignorance of the laws of chemical affinity. It is blending a chemical combination with mixture and drawing conclusions accordingly; certainly a most absurd and fatal mistake.

As pure oxygen would be too stimulating for respiration, we find it in the atmosphere diluted with nitrogen, an inert gas, simply in a state of mixture, the nature of neither being essentially changed. In like manner oxygen and hydrogen when merely mixed retain their gaseous forms and elemental properties, and while simply in a state of mixture are exceedingly inflammable and explosive, but when these two gases are chemically combined the result of that chemical union is water, which is entirely antagonistic in its properties to either of its elements. Therefore, we recognize, in this, the universal law of chemistry that whenever two elements are united by chemical affinity, the properties of both are changed and the result is a third substance differing from either; and that the elements in such union cannot act in their individual capacity till a positive decomposition is effected. Therefore, it is perfectly absurd to suppose that the blood is appropriating oxygen for the simple reason that we are inhaling something of which oxygen is a chemical constitutent. Take for further illustration these two gases—nitrogen and oxygen—in their five different proportions, chemically combined,—nitrous oxide, (N. O.), nitric oxide, (N. O.2), hyponitrous acid, (N. O.3), nitrous acid, (N. O.4), nitric acid, (N. O.5). Now bearing in mind distinctly the nature and properties of these elemental substances, or when they are only mixed as in the atmosphere and note the wonderful changes wrought by these chemical combinations.

While one proportion of nitrogen and one of oxygen form the exhilarating protoxide of nitrogen or laughing gas, two proportions of oxygen and one of nitrogen constitute nitric oxide gas, one inspiration of which would destroy life almost instantly; five proportions of oxygen to one of nitrogen form nitric acid, (aqua fortis) which cannot be tolerated a single moment by any part of the human system. Now, if it were true that the more oxygen we combine with nitrogen, the more exhilarating and healthful is the result, then, according to this logic, nitric oxide should be twice as exhilarating and healthful as nitrous oxide or laughing gas and nitric acid (aqua fortis) five times. Therefore, the logic is irresistible and the conclusion inevitable as we have demonstrated, that oxygen to be available for the support of respiration and life, must be free as in the atmosphere, and that any quantity of oxygen in chemical union with other elementary substances can avail nothing in the support of life; and that the pure atmospheric air is the only element on God's earth that will support respiration and life, and hence, is absolutely essential to an anæsthetic. And since any other elements administered instead of the pure air, always endanger health and life, the use of nitrous oxide or laughing gas, carbonic oxide and other gases for the pretended purpose of producing anæsthesia should be prohibited by law.

That pure atmospheric air is nature's only element for respiration to sustain animal or vegetable life, has been repeatedly demonstrated. When nitrous oxide gas is inhaled, it enters the blood as nitrous oxide and is eliminated as nitrous oxide. As Dr. L. Trumbull, in his Manual of Anæsthetic Agents, says: "Latterly it has been disproved both by experiment and observation, i. e., the theory which for a time prevailed in the United States that nitrous oxide acts upon the blood as an oxygenating agent, no experimental proof has yet been furnished that nitrous oxide is decomposed in the blood or forms chemical combinations with it. It enters into the blood as nitrous oxide and as such is eliminated."

As early as 1863, Dr. C. W. Foster, a dentist of high repute in Massachusetts, made a severe onslaught on the "high priests of this Modock" in the following language, "When I say nitrous oxide is not an anæsthetic, *per se*, I mean strictly so. Nitrous oxide may be given pure and with the greatest caution, but that does not affect the statement just made; the principle is the same." And then he adds, "That nitrous oxide supports combustion in the body is seen in the remarkable exhilaration of oxidation at exhibitions, etc. That it cannot support respiration or life but a few moments is seen in that frightful gasping of the patient, growing deeper and faster as he dies for want of air. These are the symptoms that precede the stupor and insensibility of this wondrous anæsthetic."

Drs. Joylet and Blanch, in the Archives de Physiologe of July, 1879, give as the result of their extensive experiments that "The protoxide of nitrogen, chemically pure, is not able to sustain respiration in animals any more than in plants, the combustion (in which respiration consists) not being sufficiently energetic to decompose the nitrous oxide gas.

"Breathed pure by animals the protoxide of nitrogen is an asphyxiating gas which produces death with all the usual signs of asphyxia by strangulation or by respiration of the inert gases (nitrogen and hydrogen) and in almost the same time. Breathed pure, if the nitrous oxide produces anæsthesia it is by privation of oxygen from the blood, insensibility showing itself when the arterial blood commences to have only two or three per cent. of oxygen. The arterial blood is then very black and contains thirty or forty per cent. protoxide of nitrogen. Animals are able by breathing an atmosphere of protoxide of nitrogen and oxygen in the proportion of the gases in the air to live, the nitrous oxide replacing the nitrogen without producing troubles of insensibility.

"The arterial blood then contains thirty to thirty five per cent. of protoxide of nitrogen. Birds plunged in a similar confined atmosphere, behave like those placed under a bell-jar of the same capacity containing air, and die after having nearly equally, consumed the oxygen in the receiver and formed as much carbonic acid. The protoxide of nitrogen being an irrespirable gas, and possessing none of the anæsthetic properties that have been attributed to it, its employment can only be dangerous and should be under that title prescribed from medical practice."

Dr. H. Lyman in his extensive and thorough work on "Artificial Anæsthesia and Anæsthetics," page 318, says: "Nitrous oxide does not enter into any chemical combination with the elements of the body, but is simply dissolved in the blood, hence its speedy entrance and departure from the organism. Again, nitrous oxide is not decomposed in the blood, consequently it cannot replace oxygen or yield oxygen for the respiration of the tissues."

A committee appointed by the British Medical Association reported to the same effect in the British Medical Journal of January, 1879, to which we invite the reader's attention.

Many other eminent authorities might be quoted to the effect that nitrous oxide is as much of an asphyxiating agent as are the inert gases, nitrogen and hydrogen, and that it will produce asphyxia and death in about the same time; but we will conclude with the following "experiments with nitrous oxide and carbonic acid," as reported in the *Odontographic Journal*, Vol. 2, No. 4, Jaunuary, 1882: "At a union meeting of two of the District Societies of Western New York, held at Buffalo, the latter part of October last, Dr. W. C. Barrett, assisted by Dr. A. P. Southwick, performed a number of experiments on small animals, using for that purpose nitrous oxide and carbonic acid. The apparatus consisted of a cylinder, a larger one of carbonic acid, and a covered glass receiver, twelve to fifteen inches in depth. Near the edge of the cover was a small opening through which, by means of rubber tubing, the gases were conveyed to the confined animals, and through which also their exhalations were permitted to escape into the room. The method followed in each experiment, was to place the animal in the receiver, adjust the cover, pass the tubing through the opening above mentioned, and then turn on the gas, the time being taken at the moment of admission of the gas, and again when life was pronounced extinct.

"The following table gives the kind of animal experimented on, the gas used, the result of the experiment, and the time consumed:

| | | | | | | | | | |
|---|---|---|---|---|---|---|---|---|---|
| 1. | Pigeon, | (common) | carbonic acid, | death, | 1 | min. | 20 | sec. | |
| 2. | " | " | nitrous oxide, | " | 1 | " | 20 | " | |
| 3. | " | (tumbler) | carbonic acid, | " | 1 | " | 10 to 25 | " | * |
| 4. | " | " | nitrous oxide, | " | 1 | " | 30 | " | |
| 5. | Rabbit, | | carbonic acid, † | " | 1 | " | 25 | " | |
| 6. | " | | nitrous oxide, | " | 1 | " | 40 | " | |
| 7. | Mouse, | | carbonic acid, | " | 1 | " | 10 | " | |

Artifical respiration was resorted to in all cases except "7", but without effect except in "5".

Thus having proven both from the very *best* of authority and the logic of facts, in demonstration of the laws of chemical affinity, that oxygen,—the life giving principle,—chemically united with any other elemental substance, cannot support life, that when taken in its pure state, it is too active and stimulating for respiration and that, when simply mixed with nitrogen as in the atmosphere, it will support respiration and life, we have *unequivocally established* the fundamental or *bedrock* principle, that there can be no anæsthetic without a proper admixture of atmospheric air with a suitable narcotic.

The definition sometimes given, that "anæsthesia is the absence of sensation," is not sufficiently explicit; nor yet is it true. For in death there is an absence of sensation and one who is dead cannot be said to be anæsthetized, for there is an absence of life as well as of sensation.

Again, there is also, in that state or condition called syncope, an absence of sensation but there is also an absence of the forces and functions of life brought about by an interruption of circulation. Nor, yet, can this condition be in any sense called anæsthesia any more than asphyxia or death can be thus named. As we have already demonstrated, insensibility and unconsciousness are the result of asphyxia as well as of anæsthesia. But that peculiar condition of the system, so much desired for surgical operations, is an absence of pain with the continuation of the forces and functions of life, and that we properly name anæsthesia. Hence, no scientist should be so stupid as to confound anæsthesia with asphyxia, or as to make little or no distinction between them.

Anæsthesia is a perfectly safe condition for surgery. But asphyxia, syncope or coma are *positively dangerous* to

* Disagreement on the part of time keepers.

† Pronounced dead in the time given, but subsequently restored to normal condition by means of artificial respiration.

life. In fact, that which is breathed in lieu of the atmosphere, that produces asphyxia, antagonizes life from the first inspiration; while asphyxia itself is a very near approach to death, just so near that when the patient has continued in that condition long enough that the affinity of the blood for oxygen has so weakened that it fails to take up the oxygen from the atmosphere, the patient is irrevocably gone.

While it is true that narcotics may be administered in such a manner and in such large percentage as to induce a suspension of the vital forces, and thus produce asphyxia or syncope, yet it is more than equally true that none of these dangerous results follow when a proper admixture of pure air with a suitable narcotic,—diluted and attenuated in such a manner and in such a percentage as to meet the wants and condition of every patient in the various stages of anæsthesia,—is administered by a competent person with suitable apparatus.

